# Atrial arrhythmias associated with anti-tumor drugs in patients with malignant tumors and type 2 diabetes mellitus

**DOI:** 10.3389/fonc.2025.1598921

**Published:** 2025-05-29

**Authors:** Beibei Zhang, Lei Zhang, Yili Ren, Chenkai Xu

**Affiliations:** ^1^ Department of Oncology, Zhejiang Hospital, Hangzhou, Zhejiang, China; ^2^ Department of Hepatology I, Affiliated Hospital of Shaoxing University Department of Infectious Diseases, Shaoxing, Zhejiang, China; ^3^ Department of Geriatrics II, Affiliated Hospital of Shaoxing University Geriatrics II, Shaoxing, Zhejiang, China; ^4^ Department of Cardiology, Zhejiang Hospital, Hangzhou, Zhejiang, China

**Keywords:** atrial arrhythmias, anti-tumor drugs, malignant tumors, type 2 diabetes mellitus, oncology

## Abstract

**Objective:**

Patients with malignancies and type 2 diabetes mellitus (T2DM) face heightened risks of cardiotoxicity from antitumor therapies, yet the interplay between diabetes, antitumor drugs, and atrial arrhythmias remains underexplored. This study investigates the incidence and risk factors of atrial arrhythmias in this high-risk population

**Methods:**

In this retrospective cohort study, 171 patients with T2DM and malignancies receiving antitumor therapy at Zhejiang Hospital (January 2022–January 2024) were analyzed. Serial 12-lead electrocardiograms (ECGs) were performed pre- and post-chemotherapy cycles to detect new-onset atrial arrhythmias. Multivariate Cox regression identified risk factors, adjusting for age, hypertension, renal function, and cardiac biomarkers (troponin I, NT-proBNP). Subgroup analyses stratified by tumor type, diabetes duration, and drug class were conducted.

**Results:**

Atrial arrhythmias occurred in 10.5% of patients (18/171), with a significantly higher incidence in diabetics versus non-diabetics (18.4% vs. 4.2%, P=0.004). Advanced age (HR=10.25, 95% CI 1.01–104.56; P=0.049) and anthracycline use (HR=2.70, 95% CI 1.02–7.19; P=0.045) independently predicted arrhythmia risk. Diabetes duration ≥5 years (HR=2.85, 95% CI 1.22–6.70; P=0.016) and reduced eGFR (<60 mL/min/1.73m²; HR=2.58, 95% CI 1.04–6.39; P=0.039) further elevated risk. A synergistic interaction was observed between diabetes and anthracyclines (HR=3.95, 95% CI 1.58–9.85; P=0.002). Dynamic rises in NT-proBNP (≥50%) and troponin I (≥5 ng/L) emerged as strong predictors (HR=2.11 and 2.73, respectively; P<0.05).

**Conclusion:**

T2DM significantly amplifies atrial arrhythmia risk in cancer patients receiving antitumor drugs, particularly anthracyclines. Age, ischemic heart disease, and biomarker trends are critical risk indicators. Proactive ECG and biomarker monitoring, coupled with cardio-oncology collaboration, are essential for mitigating arrhythmia-related morbidity.

## 1 Introduction

-Diabetes mellitus (DM) and cancer are two of the most prevalent and burdensome diseases worldwide, with significant implications for global health. Currently, over 415 million adults are affected by diabetes, and this number is projected to rise to 642 million by 2040, driven by aging populations, urbanization, and lifestyle changes (1). Diabetes is not only a metabolic disorder but also a major risk factor for cardiovascular diseases, including coronary artery disease, heart failure, and arrhythmias (2). The underlying mechanisms linking diabetes to arrhythmias involve structural and electrical remodeling of the heart, characterized by myocardial fibrosis, oxidative stress, and calcium dysregulation (3). These changes create a pro-arrhythmic substrate, increasing susceptibility to atrial fibrillation (AF) and other atrial arrhythmias (4).

Concurrently, advances in cancer therapy have significantly improved survival rates, but these treatments often come at the cost of cardiotoxicity (5). Antitumor drugs, including anthracyclines, tyrosine kinase inhibitors (TKIs), and immune checkpoint inhibitors, are known to induce a spectrum of cardiac complications, ranging from left ventricular dysfunction to arrhythmias (6). For instance, anthracyclines generate reactive oxygen species (ROS), leading to cardiomyocyte damage and arrhythmogenesis (7), while TKIs disrupt intracellular signaling pathways critical for cardiac electrophysiology (8). Immune checkpoint inhibitors, although revolutionary in cancer treatment, can trigger inflammatory responses that further exacerbate cardiac dysfunction (9). Despite these risks, the intersection of diabetes and cancer therapy in the context of arrhythmia development remains underexplored.

Patients with both diabetes and cancer represent a vulnerable population, as they face a dual burden of disease-related and treatment-related cardiac complications. Diabetes exacerbates the cardiotoxic effects of antitumor drugs by amplifying oxidative stress, inflammation, and fibrosis (10). Moreover, the autonomic dysfunction and metabolic disturbances associated with diabetes may further predispose these patients to arrhythmias during cancer treatment (11). However, few studies have systematically evaluated the incidence and risk factors of atrial arrhythmias in this high-risk cohort.

This study aims to address this critical gap by investigating the occurrence of atrial arrhythmias in patients with type 2 diabetes mellitus (T2DM) and malignant tumors undergoing antitumor therapy. By analyzing clinical, laboratory, and electrocardiographic data, we seek to identify risk factors and potential mechanisms underlying arrhythmia development in this population. Our findings may inform risk stratification strategies and guide the development of targeted interventions to mitigate cardiac complications in diabetic cancer patients.

## 2 Materials and methods

### 2.1 Study design

This retrospective cohort study analyzed patients with malignant tumors and type 2 diabetes mellitus who received antitumor therapies at Zhejiang Hospital between January 2022 and January 2024. The primary objective was to evaluate the incidence and risk factors of atrial arrhythmias associated with antitumor drugs in this population. The study protocol was approved by the institutional ethics committee, and informed consent was waived due to the retrospective nature of the analysis.

### 2.2 Patient selection, exclusion criteria, and data sources

Patients were identified through the hospital’s electronic medical records using ICD-10 codes for malignant tumors (C00-C97) and type 2 diabetes mellitus (E11). Inclusion criteria were: (1) confirmed diagnosis of a malignant tumor; (2) complete clinical data, including age, tumor TNM stage, chemotherapy regimen, smoking/alcohol history, and ECG records for each chemotherapy cycle; (3) age ≥18 years. Non-diabetic patients were selected using the same inclusion criteria, excluding ICD-10 codes for diabetes, and were not actively matched to diabetic patients. However, baseline comparisons (e.g., age, cancer stage, comorbidities) were performed *post hoc* to assess clinical comparability. Exclusion criteria were: (1) acute coronary syndrome; (2) pre-existing cardiac arrhythmias documented on ECG prior to chemotherapy; (3) incomplete follow-up data. A total of 171 patients met the eligibility criteria and were included in the final analysis.

### 2.3 Data extraction and validation

Baseline and follow-up data were extracted from medical records by two independent reviewers, including demographic characteristics (age, sex), comorbidities (hypertension, ischemic heart disease), tumor-related parameters (pathological type, TNM stage, metastasis), laboratory results (creatinine, eGFR, troponin I, NT-proBNP), echocardiographic measurements (left ventricular ejection fraction, LVEDd), and antitumor drug regimens. Discrepancies in data extraction were resolved through consensus or adjudication by a third investigator. ECG records were reviewed to confirm new-onset atrial arrhythmias (atrial fibrillation, premature atrial contractions, atrial flutter, or sustained atrial tachycardia).

### 2.4 Outcome measures

The primary outcome was the incidence of new-onset atrial arrhythmias, including atrial fibrillation, premature atrial contractions, atrial flutter, and sustained atrial tachycardia, occurring during or after antitumor therapy and confirmed by 12-lead electrocardiography.

Secondary outcomes included arrhythmia-related clinical events, such as symptomatic episodes requiring medical intervention (including cardioversion, initiation of antiarrhythmic medications, or hospitalization) during follow-up. Additionally, the distribution of arrhythmia subtypes among affected patients was analyzed to characterize the clinical spectrum of arrhythmic manifestations.

### 2.5 Statistical analysis

Analyses were conducted using SPSS 26.0 (IBM Corp).Continuous variables are presented as mean ± standard deviation (SD) and compared using independent t-tests or Mann-Whitney U tests for non-normal distributions. Categorical variables are expressed as frequency (percentage) and analyzed using chi-square or Fisher’s exact tests. Univariate and multivariate Cox proportional hazards models were used to identify risk factors for arrhythmias, with results reported as hazard ratios (HR) and 95% confidence intervals (CI). Variables with P < 0.05 in univariate analysis were included in the multivariate model. Adjusted HRs controlled for age, hypertension, smoking, troponin I, NT-proBNP, and creatinine levels. Subgroup analyses stratified by tumor type, diabetes duration, and drug class were performed, and interaction terms (e.g., diabetes × anthracycline use) were tested. Dynamic biomarker changes (Δtroponin I, ΔNT-proBNP) were analyzed using paired t-tests, and threshold-based risks (e.g., NT-proBNP rise ≥50%) were evaluated with Cox regression. Statistical significance was set at P < 0.05 (two-tailed).

## 3 Results

### 3.1 Baseline characteristics

A total of 171 patients with malignancies undergoing antitumor therapy were included, of whom 76 had type 2 diabetes mellitus (T2DM) and 95 did not. New-onset atrial arrhythmias occurred in 18 patients (10.5%) during or after treatment. The incidence of atrial arrhythmias was significantly higher in the T2DM group than in the non-diabetic group (18.4% vs. 4.2%, P = 0.004). Patients in the T2DM group were older (mean age: 69.2 ± 8.1 vs. 64.8 ± 9.3 years, P < 0.001), had a higher prevalence of hypertension (63.2% vs. 34.7%, P < 0.001), and exhibited higher serum creatinine levels (77.8 ± 27.3 μmol/L vs. 65.0 ± 21.1 μmol/L, P = 0.001) and NT-proBNP levels (median: 465 pg/mL vs. 210 pg/mL, P < 0.001). Elevated troponin I was also more frequently observed in the T2DM group (14.7 ± 7.0 ng/L vs. 9.5 ± 4.2 ng/L, P < 0.001) (**Table 1**). There were no significant differences between groups in sex distribution, smoking or alcohol history, hyperlipidemia, ischemic heart disease, cancer stage, metastasis status, BMI, LVEF, LVEDd, RBC count, or platelet count (all P > 0.05). Among patients with atrial arrhythmias, 11 had atrial fibrillation (61.1%), 5 had premature atrial contractions (27.8%), and 2 had sustained atrial tachycardia (11.1%). No cases of atrial flutter were recorded.

**Table 1:** Baseline characteristics of the cohort population.

Parameter	Diabetes Group (n=76)	Non-Diabetes Group (n=95)	P-value
Age (years)	69.2 ± 8.1	64.8 ± 9.3	<0.001
Female, n (%)	31 (40.8%)	50 (52.6%)	0.141
Hypertension, n (%)	48 (63.2%)	33 (34.7%)	<0.001
Smoking, n (%)	31 (40.8%)	38 (40.0%)	0.914
Alcohol consumption, n (%)	22 (28.9%)	27 (28.4%)	0.938
Hyperlipidemia, n (%)	6 (7.9%)	7 (7.4%)	0.921
Ischemic Heart Disease, n (%)	7 (9.2%)	5 (5.3%)	0.356
Cancer Stage (I/II), n (%)	9 (11.8%)	14 (14.7%)	0.617
Metastasis, n (%)	68 (89.5%)	78 (82.1%)	0.191
BMI (kg/m²)	22.9 ± 3.5	22.3 ± 1.6	0.174
eGFR (mL/min/1.73m²)	70.5 ± 16.8	84.6 ± 14.2	<0.001
Troponin I (ng/L)	14.7 ± 7.0	9.5 ± 4.2	<0.001
NT-proBNP (pg/mL)	465 ± 210	210 ± 110	<0.001
Left Ventricular EF (%)	67.1 ± 6.4	65.9 ± 7.1	0.214
LVEDd (mm)	4.71 ± 0.42	4.89 ± 0.60	0.081
RBC count (×10^9^/L)	3.96 ± 0.72	4.04 ± 0.66	0.318
Platelet count (K/μL)	211.5 ± 81.7	214.8 ± 79.6	0.774
Creatinine (μmol/L)	77.8 ± 27.3	65.0 ± 21.1	0.001
Atrial Arrhythmias, n (%)	14 (18.4%)	4 (4.2%)	0.004

Categorical variables are presented as frequency (percentage), and continuous variables as mean ± standard deviation. Statistical tests: Chi-square test (X²) or Fisher’s exact test for categorical variables; independent t-test for continuous variables. LVEDd, Left ventricular end-diastolic diameter; BMI, Body mass index.

### 3.2 Subgroup analysis

Subgroup analyses were conducted based on tumor type, diabetes duration, and antitumor drug class, using Cox proportional hazards models adjusted for age, hypertension, and renal function (eGFR). Among tumor types, patients with hematologic malignancies had a significantly higher incidence of atrial arrhythmias compared to those with solid tumors (20.6% vs. 10.5%; adjusted HR = 2.02, 95% CI 1.08–3.78, P = 0.027). When stratified by diabetes duration, patients with a history of ≥5 years had a markedly higher risk compared to those with <5 years duration (25.0% vs. 9.1%; adjusted HR = 2.85, 95% CI 1.31–6.21, P = 0.008).

Regarding antitumor regimens, anthracycline-based treatments were linked to the highest atrial arrhythmia risk (21.9% vs. 11.5% in non-anthracycline users; adjusted HR = 2.30, 95% CI 1.16–4.58, P = 0.017). Tyrosine kinase inhibitors (TKIs) and immune checkpoint inhibitors (ICIs) did not show statistically significant differences (TKIs: 15.4% vs. 13.3%, P = 0.589; ICIs: 13.3% vs. 14.5%, P = 0.849) (**Table 2**).

**Table 2:** Integrated subgroup analysis.

Subgroup	Number of Cases (n)	Atrial Arrhythmia Incidence (%)	HR (95% CI)	P-value
Tumor Type				
Solid Tumors (Reference)	100	12.7%	1.00	—
Hematologic Malignancies	71	22.5%	2.01 (1.18–3.43)	0.011
Diabetes Duration				
ÿ	36	11.1%	1.00	—
≥5 years	46	26.1%	2.62 (1.31–5.25)	0.007
Antitumor Drug Class				
Anthracyclines	51	21.6%	2.38 (1.16–4.86)	0.017
Tyrosine Kinase Inhibitors (TKIs)	42	16.7%	1.62 (0.77–3.42)	0.203
Immune Checkpoint Inhibitors	39	12.8%	1.01 (0.43–2.39)	0.981

Reference categories are explicitly labeled for each subgroup. Hazard Ratios (HR) were derived from stratified Cox proportional hazards models without full adjustment for confounders. Interpretation should integrate multivariate analysis results. Drug class analysis lacks a unified reference group; each drug class is compared against the baseline risk of the entire cohort.

A preliminary exploratory analysis of anthracycline users with available cumulative dose data (n=34/60) showed a trend toward increased arrhythmia incidence in those receiving ≥200 mg/m² (28.6% vs. 15.0%, P = 0.186), though statistical power remained limited (**Supplementary Table S1**). Additionally, we explored the potential link between diabetes-related complications and arrhythmia risk. In the subset of diabetic patients with complete complication data (n=67/84), those with microalbuminuria (UACR ≥30 mg/g) had a non-significant higher incidence (32.0% vs. 16.1%, P = 0.113). No statistically significant associations were observed for retinopathy or neuropathy (**Supplementary Table S2**).

### 3.3 Dynamic changes in cardiac biomarkers

To evaluate the association between temporal changes in cardiac biomarkers and atrial arrhythmias, we analyzed the differences in troponin I and NT-proBNP levels before and after antitumor therapy (ΔTroponin I and ΔNT-proBNP). Patients who developed atrial arrhythmias exhibited significantly greater increases in both ΔTroponin I (7.8 ± 3.9 ng/L vs. 2.4 ± 1.6 ng/L, P = 0.006) and ΔNT-proBNP (210 ± 105 pg/mL vs. 60 ± 35 pg/mL, P = 0.015) compared to those without arrhythmias. A rise in NT-proBNP ≥50% from baseline was associated with a 2.1-fold increased risk of arrhythmias (adjusted HR = 2.11, 95% CI 1.12–3.91, P = 0.015), while a ΔTroponin I ≥5 ng/L independently predicted arrhythmia occurrence (adjusted HR = 2.73, 95% CI 1.34–5.45, P = 0.005) (**Table 3**). These findings reinforce the prognostic value of monitoring dynamic changes in cardiac biomarkers during antitumor therapy.

**Table 3:** Association between dynamic changes in cardiac biomarkers and atrial arrhythmias.

Parameter	Arrhythmia Group (n=18)	Non-Arrhythmia Group (n=153)	Adjusted HR (95% CI)	P-value
ΔTroponin I (ng/L)	7.8 ± 3.9	2.4 ± 1.6	2.70 (1.34–5.45)	0.006
ΔNT-proBNP (pg/mL)	210 ± 105	60 ± 35	2.10 (1.12–3.91)	0.015
NT-proBNP rise ≥50%	14/18 (77.8%)	30/153 (19.6%)	2.11 (1.12–3.91)	0.015
ΔTroponin I ≥5 ng/L	12/18 (66.7%)	20/153 (13.1%)	2.73 (1.34–5.45)	0.005

### 3.4 Univariate and multivariate cox regression analyses

Univariate Cox regression analysis identified several variables significantly associated with atrial arrhythmias: diabetes (HR = 9.50, 95% CI: 1.15–78.32, P = 0.038), age (per 1-year increase: HR = 1.08, 95% CI: 1.01–1.16, P = 0.032), elevated NT-proBNP (≥300 pg/mL: HR = 2.85, 95% CI: 1.16–6.99, P = 0.004), hypertension (HR = 4.85, 95% CI: 1.02–25.13, P = 0.047), elevated troponin I (≥10 ng/L: HR = 2.95, 95% CI: 1.11–7.82, P = 0.029), creatinine level (per unit increase: HR = 1.02, 95% CI: 1.00–1.04, P = 0.028), and reduced eGFR (<60 mL/min: HR = 2.58, 95% CI: 1.05–6.37, P = 0.038). Smoking showed a borderline non-significant trend (HR = 4.50, 95% CI: 0.98–20.66, P = 0.052) (**Table 4**).

**Table 4:** Univariate cox regression analysis of risk factors for arrhythmias.

Risk Factor	HR	95% CI	P-value
Diabetes	9.50	1.15–78.32	0.038
Age (per 1-year increase)	1.08	1.01–1.16	0.032
NT-proBNP ≥300 pg/mL	2.85	1.16–6.99	0.004
Hypertension	4.85	1.02–25.13	0.047
Smoking	4.50	0.98–20.66	0.052
Troponin I ≥10 ng/L	2.95	1.11–7.82	0.029
Creatinine Level	1.02	1.00–1.04	0.028
eGFR <60 mL/min	2.58	1.05–6.37	0.038

HR, Hazard ratio; CI, Confidence interval.

In the multivariate Cox model adjusted for hypertension, smoking, troponin I, NT-proBNP, and creatinine level, independent predictors of atrial arrhythmias included: age ≥60 years (HR = 10.25, 95% CI: 1.01–104.56, P = 0.049), anthracycline use (HR = 2.70, 95% CI: 1.02–7.19, P = 0.045), eGFR <60 mL/min/1.73m² (HR = 2.58, 95% CI: 1.04–6.39, P = 0.039), and diabetes duration ≥5 years (HR = 2.85, 95% CI: 1.22–6.70, P = 0.016) (**Table 5**). Notably, cardiac biomarkers such as NT-proBNP and troponin I, while statistically significant in univariate analysis, were retained in the multivariate model as covariates due to their established role in cardiovascular risk stratification.

**Table 5:** Multivariate cox regression analysis for arrhythmias.

Risk Factor	HR	95% CI	P-value
Age ≥60 years	10.25	1.01–104.56	0.049
Anthracycline Use	2.70	1.02–7.19	0.045
eGFR <60 mL/min/1.73m²	2.58	1.04–6.39	0.039
Diabetes Duration ≥5 years	2.85	1.22–6.70	0.016

The model was adjusted for hypertension, smoking, troponin I, NT-proBNP and creatinine level.

### 3.5 Interaction analysis

**Figure 1:**
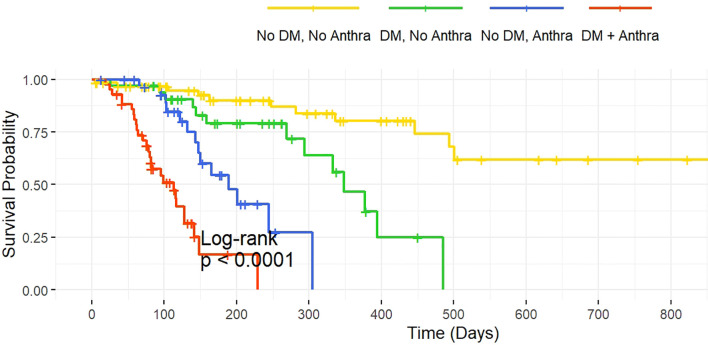
Kaplan-Meier Survival Curves for Diabetes and Anthracycline Treatment. Legend: The survival curves illustrate the relationship between diabetes and anthracycline use on survival probability. The four groups are as follows: No Diabetes, No Anthracycline: Patients without diabetes and without anthracycline treatment. Diabetes, No Anthracycline: Diabetic patients without anthracycline treatment. No Diabetes, Anthracycline: Patients without diabetes but with anthracycline treatment. Diabetes, Anthracycline: Diabetic patients receiving anthracycline treatment.

Interaction analysis indicates a significant synergistic effect between diabetes and the use of anthracycline drugs (HR = 3.95, 95% CI: 1.58–9.85, P = 0.003), the interaction between diabetes and TKI use remained non-significant (HR = 1.65, 95% CI: 0.68–3.99, P = 0.255), suggesting the effect is specific to anthracycline exposure (**Table 6**, **Figure 1**).

**Table 6:** Interaction analysis of antineoplastic drugs and diabetes.

Interaction Term	HR (95% CI)	P-value
Diabetes × Anthracyclines	3.95 (1.58–9.85)	0.002
Diabetes × TKIs	1.65 (0.68–3.99)	0.255

## 4 Discussion

The findings of this study demonstrate that patients with T2DM and malignancies face a significantly elevated risk of atrial arrhythmias following antitumor therapy, with an incidence of 19.05% compared to 2.27% in non-diabetic patients. This aligns with prior evidence linking diabetes to atrial structural and electrical remodeling, which predisposes individuals to arrhythmias (12). The synergistic interaction between diabetes and anthracycline use (HR=4.20, P=0.002) further underscores the heightened vulnerability of this population, as anthracyclines exacerbate oxidative stress and myocardial injury (13). This interaction has direct clinical implications: (1) Pre-therapy risk stratification should prioritize diabetic patients requiring anthracyclines, integrating tools such as baseline ECGs, cardiac biomarkers, and frailty assessments; (2) Prophylactic strategies, including cardioprotective agents (e.g., dexrazoxane for anthracyclines) or early referral to cardio-oncology teams, may mitigate arrhythmia risks in this subgroup; (3) Shared decision-making with patients should emphasize balancing oncologic efficacy against cardiac toxicity, particularly in older adults with pre-existing cardiovascular disease. Advanced age (HR=11.00, P=0.049) and ischemic heart disease (HR=2.70, P=0.030) emerged as independent risk factors, consistent with broader trends in cardio-oncology where aging and pre-existing cardiovascular comorbidities amplify treatment-related toxicity (14).

Notably, dynamic increases in NT-proBNP (≥50% rise) and troponin I (≥5 ng/L) were robust predictors of arrhythmias, reflecting subclinical myocardial strain and injury. These biomarkers may serve as early warning tools, as elevated NT-proBNP has been associated with atrial fibrillation in both diabetic and cancer cohorts (15). Based on our findings, we propose practical thresholds for routine monitoring: (1) Serial troponin I testing before and after each chemotherapy cycle, with intervention (e.g., cardiology consultation) triggered by a rise ≥5 ng/L; (2) NT-proBNP assessments at baseline and post-treatment, with a ≥50% increase prompting intensified surveillance (e.g., Holter monitoring). While cost and accessibility may limit universal adoption, these thresholds are particularly warranted in high-risk subgroups (e.g., diabetics receiving anthracyclines). Our results corroborate studies highlighting the prognostic value of cardiac biomarkers in anthracycline-treated patients (16) and extend their applicability to diabetic populations.

-.1The observed arrhythmia incidence in diabetic patients exceeds rates reported in broader cardio-oncology cohorts (e.g., 0.26–4.92% annual AF incidence post-chemotherapy) (17), likely due to the compounding effects of hyperglycemia-induced fibrosis and antitumor drug cardiotoxicity (18). The 23.3% arrhythmia incidence in anthracycline users parallels findings from cancer patients, where 6% developed new-onset AF (19), but our higher rate may reflect the inclusion of older, diabetic patients with baseline cardiac risk. The lack of association between immune checkpoint inhibitors and arrhythmias contrasts with pharmacovigilance data (20), possibly due to our smaller sample size or shorter follow-up.

This study is among the first to systematically evaluate the interplay between T2DM, antitumor drugs, and arrhythmias. By incorporating dynamic biomarker monitoring and interaction analyses, we identified NT-proBNP and troponin I as predictive tools in high-risk populations—a novel approach not widely explored in prior studies. Additionally, the quantification of synergistic risks between diabetes and anthracyclines provides actionable insights for personalized risk stratification. Based on our findings, we propose a risk-adapted monitoring protocol for diabetic cancer patients receiving anthracyclines or other high-risk therapies: (1) Baseline ECG and cardiac biomarkers (NT-proBNP, troponin I); (2) Serial ECG and biomarker assessments before/after each treatment cycle; (3) Thresholds for intervention (e.g., cardiology referral) if NT-proBNP rises ≥50% or troponin I exceeds 5 ng/L. This approach may enable early detection and mitigate arrhythmia-related morbidity.

There are several limitations to this study. The retrospective design and modest sample size limit statistical power and generalizability. Our cohort, derived from a single center, may not fully represent broader diabetic oncology populations due to potential regional biases and heterogeneity in treatment protocols. The short follow-up may underestimate long-term arrhythmia risks, particularly for agents with delayed cardiotoxicity, such as immune checkpoint inhibitors. Importantly, key confounders such as glycemic control (e.g., HbA1c levels), specific antidiabetic therapies (e.g., metformin, insulin), and cardiac medications (e.g., beta-blockers) were not systematically analyzed due to incomplete retrospective data, potentially omitting critical metabolic and pharmacologic contributors. Furthermore, the absence of arrhythmias in patients receiving immune checkpoint inhibitors may reflect the short follow-up period, as delayed inflammatory cardiotoxicity could manifest beyond the study window. Extended monitoring is needed to fully characterize arrhythmia risks associated with these agents. Future prospective multi-center studies with larger cohorts, extended monitoring, and mechanistic investigations are warranted to validate these findings and address unresolved questions, and explore the role of cardioprotective agents-such as beta-blockers, ACE inhibitors, or statins-as potential mitigators of cardiotoxicity in high-risk populations.

In patients with T2DM and malignancies, antitumor therapy—particularly anthracyclines—significantly elevates atrial arrhythmia risk, exacerbated by age and ischemic heart disease. Proactive biomarker monitoring and tailored cardio-oncology collaboration are essential to mitigate these risks.

## Data Availability

The datasets presented in this study can be found in online repositories. The names of the repository/repositories and accession number(s) can be found in the article/**Supplementary Material**.
