# Supplementary material for: Atrial arrhythmias associated with anti-tumor drugs in patients with malignant tumors and type 2 diabetes mellitus
Source: Front Oncol. 2025 May 29;15:1598921. doi: 10.3389/fonc.2025.1598921 (PMC12158937; doi:10.3389/fonc.2025.1598921)
Supplement: Supplementary file 1 [file Table1.docx]

Supplementary Tables

**Table S1. Exploratory Analysis of Anthracycline Cumulative Dose and Arrhythmia Risk**

| Cumulative Dose (Doxorubicin-equivalent) | Number of Patients | Atrial Arrhythmia Incidence (%) | P-value |
| --- | --- | --- | --- |
| <200 mg/m² | 12 | 16.7% | 0.28 |
| ≥200 mg/m² | 6 | 33.3% |  |

Notes: Data available for 18/30 anthracycline users (60%). Statistical analysis via Fisher’s exact test due to small sample size. Incomplete retrospective records precluded formal dose-effect analysis.

**Table S2. Exploratory Analysis of Diabetic Complications and Arrhythmia Risk**

| Complication | Patients with Complication (n) | Atrial Arrhythmia Incidence (%) | P-value |
| --- | --- | --- | --- |
| Microalbuminuria (UACR ≥30 mg/g) | 14 | 35.7% | 0.22 |
| Retinopathy | 8 | 25.0% | 0.45 |
| Neuropathy | 6 | 16.7% | 0.64 |

Notes: Data available for 32/42 diabetic patients (76.2%). Statistical analysis via Fisher’s exact test due to small sample size. Retrospective documentation of complications was incomplete.
